# Prophylactic therapy for prevention of surgical site infection after extraction of third molar: An overview of reviews

**DOI:** 10.4317/medoral.25999

**Published:** 2023-07-10

**Authors:** Yiting Cao, Qian Jiang, Jingzhou Hu

**Affiliations:** 1 Department of Pediatric Dentistry, Shanghai Ninth People’s Hospital, Shanghai Jiao Tong University School of Medicine; College of Stomatology, Shanghai Jiao Tong University; National Center for Stomatology; National Clinical Research Center for Oral Diseases; Shanghai Key Laboratory of Stomatology; Shanghai Research Institute of Stomatology. Shanghai, China; 2 Department of Oral Surgery, Shanghai Ninth People's Hospital, Shanghai Jiao Tong University School of Medicine; College of Stomatology, Shanghai Jiao Tong University; National Center for Stomatology; National Clinical Research Center for Oral Diseases; Shanghai Key Laboratory of Stomatology; Shanghai Research Institute of Stomatology. Shanghai, China; 3 Department of Oral and Maxillofacial-Head and Neck Oncology, Shanghai Ninth People's Hospital, Shanghai Jiao Tong University School of Medicine; College of Stomatology, Shanghai Jiao Tong University; National Center for Stomatology; National Clinical Research Center for Oral Diseases; Shanghai Key Laboratory of Stomatology; Shanghai Research Institute of Stomatology. Shanghai, China

## Abstract

**Background:**

To compare the effect of different prophylactic therapies on prevention of surgical site infection after extraction of third molars with different degree of impaction.

**Material and Methods:**

Systematic reviews and meta-analyses evaluating the effect of different prophylactic therapies on prevention of surgical site infection after extraction of third molars were included. An electronic search was performed in PubMed, EMBASE, and the Cochrane Database of Systematic reviews. AMSTAR 2 tool was used to evaluate the confidence in results from the included reviews. Descriptive analyses were performed.

**Results:**

Six reviews were included. A significant benefit of different antibiotics to the prevention of site infection after extraction of third molars was reported. Amoxicillin/amoxicillin clavulanic acid could significantly reduce the rate of surgical site infection versus placebo. Chlorhexidine gel could significantly reduce the frequency of alveolar osteitis versus placebo.

**Conclusions:**

Based on the limited evidence, there is a significant benefit of prophylactic therapy while the comparative effect of different types of prophylactic regimes are controversial.

** Key words:**Third molar extraction, antibiotics, chlorhexidine, overview.

## Introduction

Extraction of third molars with different impaction degree has been a common practice in dental clinics. Although third molar extraction is commonly considered as a safe procedure, complications might occur during or after surgery. A previous cross-sectional study reported a post-surgery complication rate of 6.9%, including alveolitis, infection, and paresthesia of the inferior alveolar nerve (1). A recent review summarizes the frequency of post-surgery complications, including alveolitis (0.5 to 32.5%), infection (0.9 to 4.2%), postoperative bleeding (0.2 to 1.5%), as well as transient and permanent dysfunction of the inferior alveolar nerve (0.6 to 5.5%, 0.1 to 0.9%, respectively) (2). The complications during or after surgery might lead to patient discomfort, impaired oral and systematic health, and reduced quality of life (3). Thus, more attention should be paid to the prevention and management of complications following third molar extraction.

Third molars usually not fully erupt and the complexity of surgery procedure might increase the risks of surgery site infection (1). Use of prophylactic therapy has been reported to prevent surgery site infection after third molar extraction. Prescription of systematic antibiotics is a wide spread practice among dentists. A previous systematic review reports that 19 people need to be treated with antibiotics to prevent one infection following extraction of impacted third molars (4). However, inappropriate use of antibiotics has the risks of adverse reactions and antibiotic resistance (5). Thus, prophylactic antibiotic therapy is usually not indicated in healthy patients. Some authors explored to use chlorhexidine as prophylactic therapy due to broad spectrum activity, covering anaerobes, and no registered resistance. However, the benefit of chlorhexidine on prevention of infection following third molar extraction is also unclear (6,7).

Currently, several systematic reviews have addressed the effect of different prophylactic therapies on prevention of infection following third molar extraction. However, an overall critical appraisal and synthesis has not been performed. Thus, the objective of the present review is to systematically identify and critically appraise current systematic reviews on the application of prophylactic medication for the prevention of surgical site infection after extraction of third molars with different degree of impaction.

## Material and Methods

The protocol of the present review was elaborated prospectively（Supplement 1）. The present overview is reported based on the preferred reporting items for systematic reviews and meta-analyses (PRISMA) checklist (8). No ethics approval is required for this overview.

- Eligible criteria

The PICOS format research question is as follows. Patients were those who need extraction of third molars with different degree of impaction. Interventions were different types of prophylactic medication. Comparisons were types of prophylactic medication other than interventions, or placebo. Primary outcome measure was frequency of surgical site infection, including infection, dry socket, fever, lymphadenopathy, or alveolar osteitis. The study design was systematic review with or without meta-analysis.

- Search strategy

Electronic search was performed in PubMed, Embase, and the Cochrane database of systematic review. No limitation was set on language and publication time. The search was performed in January, 2023. The search strategy was (review[Title]) AND (third molar*[Title/Abstract]) AND (remov*[Title/Abstract] OR extract*[Title/Abstract] OR infect*[Title/Abstract]). Hand search was performed by screening reference lists and citing articles of key studies.

- Study selection

Two review authors performed study selection independently and in duplicate. Firstly, title/abstract of records were screened. Secondly, full articles were screened. A data mining tool Rayan (https://www.rayyan.ai) was used as a third screener to assist in study selection. Any disagreement was solved by discussion with experts. The inter-reviewer reliability (kappa correlation coefficient) of the title/abstract screening and full-text screening was 0.86 and 0.81, respectively.

- Data extraction

Data extraction was performed by two review authors independently and in duplicate. The following information was extracted: bibliometrics information (author, publication year), study methods (patient characteristics, interventions, comparison, outcome measures), information for risks of bias assessment (risk of bias tool, funding, conflict of interest), main results (type of data synthesis, effect size, 95% confidence interval, *p-value*, and heterogeneity), and main conclusions.

- Risks of bias assessment

Risks of bias of included systematic reviews was assessed by a MeaSurement Tool to Assess systematic Reviews (AMSTAR)-2 tool (9). Two review authors performed risk of bias assessment independently and in duplicate. Briefly, seven domains with 16 items were assessed, including protocol registration, adequacy of literature search, justification for excluding individual studies, risks of bias of individual studies, appropriateness of meta-analysis methods, consideration of risks of bias in result interpretation, and assessment of publication bias. The overall confidence of each systematic review was assessed as high, moderate, low, and very low with the presence of no or one non-critical weakness, more than one non-critical weakness, one critical weakness with or without one non-critical weakness, and more than one critical weakness, respectively.

- Data synthesis

Qualitative analysis was performed summarizing the frequency of surgical site infection, reported in relative or absolute frequencies as main summary measure.

## Results

- Study selection

**Figure 1:**
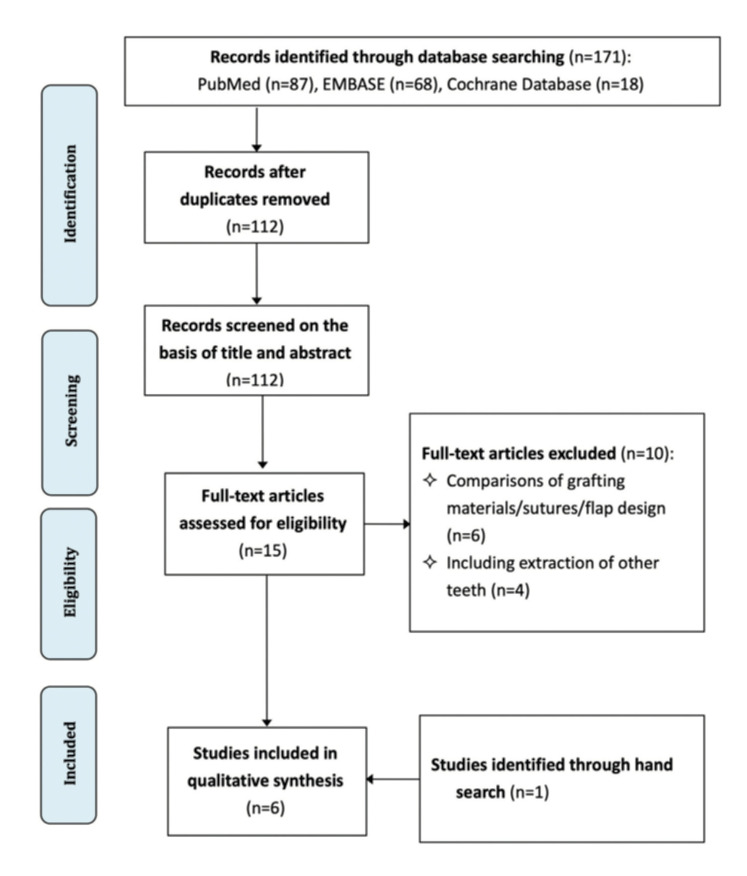
Flow diagram.

Electronic search identified 87, 68, and 18 records in PubMed, Embase, and Cochrane Database of Systematic reviews, respectively. After removal of duplicates, 112 records were screened for titles/abstracts. Fifteen studies were screened for full publication and ten were excluded. Hand search identified one study. Procedures for study selection is showed in Fig. 1. Finally, six studies were included (10-15).

- Characteristics of included studies

Characteristics of included studies are listed in Table 1. All the reviews included only randomized controlled trials (RCTs). The interventions include amoxicillin/amoxicillin clavulanic acid only (10), chlorhexidine only (15), or combination of different therapies. The comparisons include placebo alone or different therapies other than intervention. The outcome measure was incidence of infection.

**Table 1:**
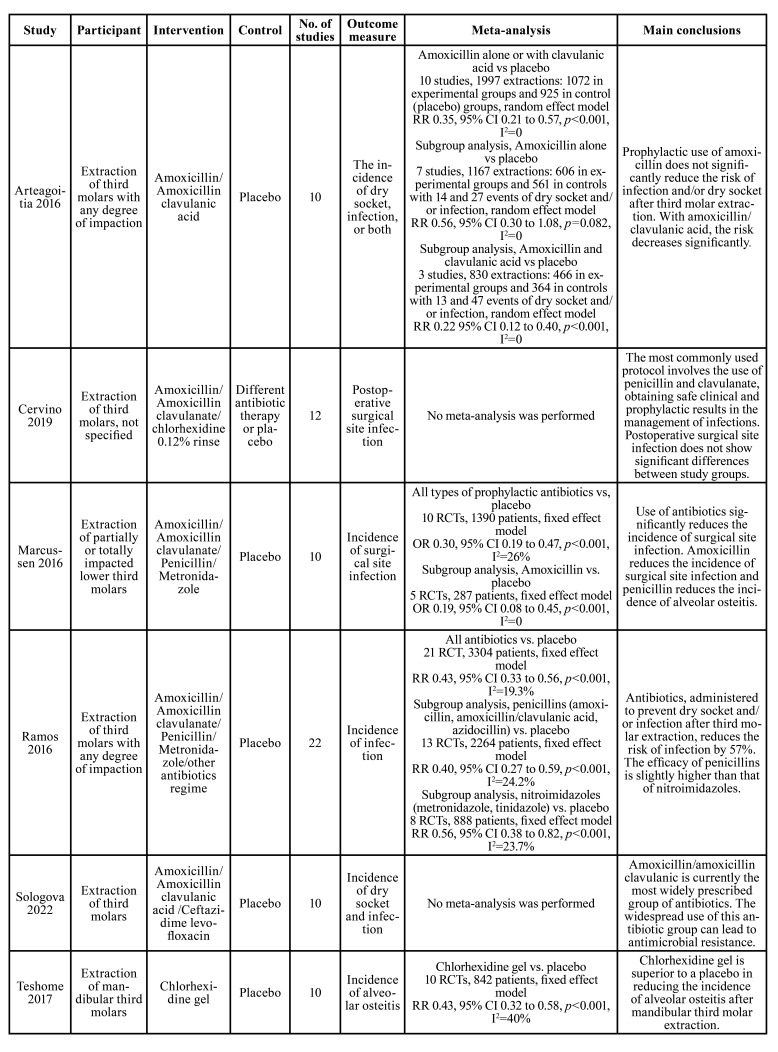
Characteristics of included reviews.

- Risks of bias of included studies

Risk of bias of included studies assessed by the AMSTAR-2 tool is listed in Table 2. Three studies were assessed as low confidence and three as very low.

-Descriptive data analysis

1 Any type of antibiotics

Two studies reported significant benefit of different antibiotics to the prevention of site infection after extraction of third molars. Marcussen *et al* (2016) included 10 RCTs with 1390 participants and reported significant benefit of different regime of antibiotics compared with placebo (OR=0.30, 95% CI 0.19 to 0.47, *p*<0.001, I2=26%). Similarly, Ramos *et al* (2016) included 21 RCTs with 3304 participants and reported significant benefit of different regime of antibiotics compared with placebo (RR=0.43, 95% CI 0.33 to 0.56, *p*<0.001, I2=19.3%). These two studies concluded that use of antibiotics significantly reduced the frequency of surgical site infection.

2 Amoxicillin/amoxicillin clavulanic acid

One study, including 10 studies and 1997 participants, focused on the effect of amoxicillin/amoxicillin clavulanic acid (10). This study reported that amoxicillin alone did not significantly reduce the rate of surgical site infection (RR=0.56, 95% CI 0.30 to 1.08, *p*=0.082, I2=0) while amoxicillin with clavulanic acid significantly reduced the rate of surgical site infection (RR=0.22, 95% CI 0.12 to 0.40, *p*<0.001, I2=0). The study also reported that 1 out of 26 patients treated with amoxicillin with or without clavulanic acid had some types of adverse events. Marcussen *et al* (2016) included different types of antibiotics and performed subgroup analysis on the effect of amoxicillin versus placebo. The result showed that amoxicillin clavulanic acid could significantly reduce the incidence of surgical site infection (5 RCTs, 287 patients, OR=0.19, 95% CI 0.08 to 0.45, *p*<0.001, I2=0).

3 Penicillin

One study performed subgroup analysis on penicillin versus placebo and reported that penicillin could significantly reduce the incidence of alveolar osteitis (2 RCTs, 188 patients, OR=0.10, 95% CI 0.03 to 0.30, *p*<0.001, I2=0).

**Table 2:**
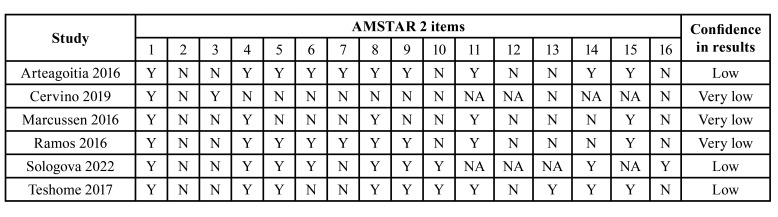
AMSTAR 2 assessment of confidence in results of systematic reviews included

4 Chlorhexidine

One study including 10 RCTs and 842 patients compared the prevention effect of chlorhexidine gel versus placebo regarding the frequency of alveolar osteitis (15). The meta-analysis showed benefit of using chlorhexidine gel (RR=0.43, 95% CI 0.32 to 0.58, *p*<0.001, I2=40%).

## Discussion

This is the first overview to systematically summarize and critically appraise the available high-level evidence on the benefit of prophylactic therapy on prevention of incidence of surgery site infection after extraction of third molar with different degree of impaction. The current evidence suggests that there is a significant benefit of prophylactic therapy while the comparative effect of different types of prophylactic regimes are controversial. It is difficult to determine which one is the best prophylactic therapy following third molar extraction.

Overall, two studies reported significant benefit of different antibiotics to the prevention of site infection after extraction of third molars (12,13). This is in accordance to a previous Cochrane systematic review, which reported that following extraction of third molars, antibiotic prophylaxis might reduce the rate of postsurgical infectious complications by 66% and reduce the risks of dry socket by 34%, respectively (4).The results indicated that 19 and 46 patients need to be treated with antibiotics to prevent one infection and dry socket, respectively. Based on the current evidence, there is significant benefit of antibiotics on the prevention of infection after third molar extraction. However, the adverse events of antibiotics should be remembered. The risk of post-surgery infection is associated with several factors, including the impaction degree and anatomical position of third molar, patient oral and systematic health, surgeon’s experience, and management of hemostasis during surgery (1,16-18). Clinicians should carefully study the patient conditions and evaluate risks of infection, before prescription of antibiotics.

Amoxicillin, alone or combined with clavulanic acid, is reported to be the most widely used prophylactic antibiotics (14). Two studies reported significant benefit of amoxicillin clavulanic acid (10,12) while the effect of amoxicillin alone was not significant (10). The results should be interpreted with cautious because of the limited sample size and very low confidence in results. In addition, the adverse events of amoxicillin/amoxicillin clavulanic acid should be addressed. Another study reported that a single preoperative oral dose of 2 g of amoxicillin significantly reduce the risks of surgical site infection (12). This study discussed that there was no evidence of development of resistance after application of a single prophylactic dosage of an antibiotic. The different results derived from different studies might be attributed to the different third molar impaction degree, surgical techniques, dose of antibiotics, and control groups (placebo or other types of antibiotics).

A significant beneficial effect of chlorhexidine gel on the prevention of alveolar osteitis was reported in one study (15). Chlorhexidine has been used in dentistry for over 40 years as an antiplaque agent, due to its high substantivity and broad antimicrobial spectrum (19). It was reported that chlorhexidine mouth rinse could prevent dry socket and bacteremia after tooth extraction (20,21). However, disadvantages of chlorhexidine mouth rinse have been reported, including impaired taste sensation, increased supragingival calculus formation, oral mucosal lesion, as well as tooth restoration and tongue staining (22-24). In addition, mouth rinse should be avoided within 24 hours after tooth extraction. Thus, application of chlorhexidine gel might be a better choice. It has been reported that chlorhexidine gel could promote wound healing after tooth extraction (19), which was in accordance with the present review.

There are some limitations of the present overview. Firstly, the overall confidence in results was low to very low, due to the inferior methodology of included systematic reviews. Secondly, there was large heterogeneity among studies, due to different jaw locations, impaction degree, surgical interventions, dosage of prophylactic therapies, and patient conditions. Thirdly, we failed to perform a quantitative analysis due to the different choice of prophylactic therapies among studies and large heterogeneity. Thus, in the present review, we failed to draw a conclusion on which therapy was better to prevent infection after third molar extraction. In the future, trials comparing different types of prophylactic therapies are recommended. Trials should apply strictly eligible criteria, high-quality methodology, validated outcome measures, and well-formatted reporting structures.

## Conclusions

The current evidence suggests that, regarding prevention of infection of third molar extraction, there is a significant benefit of prophylactic therapy while the comparative effect of different types of prophylactic regimes are controversial. Based on the limited evidence, it is difficult to determine which one is the best prophylactic therapy following third molar extraction.
